# Low Radiation Environment Switches the Overgrowth-Induced Cell Apoptosis Toward Autophagy

**DOI:** 10.3389/fpubh.2020.594789

**Published:** 2021-01-12

**Authors:** Mariafausta Fischietti, Emiliano Fratini, Daniela Verzella, Davide Vecchiotti, Daria Capece, Barbara Di Francesco, Giuseppe Esposito, Marco Balata, Luca Ioannuci, Pamela Sykes, Luigi Satta, Francesca Zazzeroni, Alessandra Tessitore, Maria Antonella Tabocchini, Edoardo Alesse

**Affiliations:** ^1^Museo Storico della Fisica e Centro Studi e Ricerche Enrico Fermi, Rome, Italy; ^2^Department of Biotechnological and Applied Clinical Sciences, L'Aquila University, L'Aquila, Italy; ^3^Istituto Superiore di Sanità, National Center for Innovative Technologies in Public Health, Rome, Italy; ^4^Istituto Nazionale di Fisica Nucleare (INFN) Sezione Roma, Rome, Italy; ^5^INFN-Gran Sasso National Laboratory, Assergi L'Aquila, Italy; ^6^Flinders Center for Innovation in Cancer, Flinders University, Adelaide, SA, Australia

**Keywords:** low radiation environment, LRE, apoptosis, autophagy, PARP1, p53

## Abstract

Low radiation doses can affect and modulate cell responses to various stress stimuli, resulting in perturbations leading to resistance or sensitivity to damage. To explore possible mechanisms taking place at an environmental radiation exposure, we set-up twin biological models, one growing in a low radiation environment (LRE) laboratory at the Gran Sasso National Laboratory, and one growing in a reference radiation environment (RRE) laboratory at the Italian National Health Institute (Istituto Superiore di Sanità, ISS). Studies were performed on pKZ1 A11 mouse hybridoma cells, which are derived from the pKZ1 transgenic mouse model used to study the effects of low dose radiation, and focused on the analysis of cellular/molecular end-points, such as proliferation and expression of key proteins involved in stress response, apoptosis, and autophagy. Cells cultured up to 4 weeks in LRE showed no significant differences in proliferation rate compared to cells cultured in RRE. However, caspase-3 activation and PARP1 cleavage were observed in cells entering to an overgrowth state in RRE, indicating a triggering of apoptosis due to growth-stress conditions. Notably, in LRE conditions, cells responded to growth stress by switching toward autophagy. Interestingly, autophagic signaling induced by overgrowth in LRE correlated with activation of p53. Finally, the gamma component of environmental radiation did not significantly influence these biological responses since cells grown in LRE either in incubators with or without an iron shield did not modify their responses. Overall, *in vitro* data presented here suggest the hypothesis that environmental radiation contributes to the development and maintenance of balance and defense response in organisms.

## Introduction

Life has evolved on Earth for more than three billion years in ecosystems characterized by different levels of environmental radiation. This abiotic factor, acting as a natural tiny but constant daily stimulus, is at the heart of the development of life and has been incorporated within the biology of organisms during evolution (1). However, very little is known about molecular mechanisms underpinning the effects of this influencing factor on living beings. To this aim, analysis of differences between two parallel biological systems, one kept in a reference radiation environment (RRE) and one in a very low radiation environment (LRE), helps to enhance knowledge in this field. During the last decades, studies performed in underground laboratories highlighted that biological models could react to background radiation changes in different ways. It was demonstrated that behavior of living systems under low radiation dose exposure can lead to interesting, and often unexpected, results. Several effects, such as genomic instability, with increase of DNA changes through generations; transgenerational effects, with hereditary alterations; bystander effect, which causes damage of healthy cells near to those irradiated, have been observed (2–4).

The peculiar location of the underground Gran Sasso National Laboratory (LNGS) of the Italian Institute of Nuclear Physics (INFN), characterized by very low-radiation conditions, makes it particularly suitable for implementing not only studies in the field of physics, such as proton decay or solar neutrino detection, but also in that of biology. In this location, shielded by more than 1400 meters of carbonate (dolomia) rock, cosmic radiation is almost completely absent (5). Furthermore, due to the nature of the rocks, Uranium and Thorium are barely detected, and the neutron flux is reduced by a factor of 10^3^ with respect to external values (6). In addition, radon concentration is kept at a very low level by an efficient ventilation system that pumps air from the outside into the laboratory.

Accordingly, the low LET components, especially photons, are predominant at LRE. On the other hand, at RRE, there are both low LET and high LET components.

Previous researches demonstrated the influence of different environmental radiation on biological systems of different origin (yeast, mouse, and human), indicating that cell cultures maintained in LRE, compared to those in RRE, developed a different biochemical response, being less preserved from DNA damage and showing reduced Reactive Oxygen Species (ROS) scavenging power (1, 7–9).

In this study, we took advantage of the Gran Sasso National Laboratory to further show the core mechanisms putatively responsible for different cellular behaviors attributable to LRE. In particular, we investigated the molecular response of the pKZ1 A11 mouse hybridoma cells (10, 11), cultured in parallel in LRE (at LNGS) and in RRE (at ISS) laboratories, to overgrowth stress conditions, in which cells undergo nutrient deprivation, hypoxia, and pH unbalance. We found that radiation environment does not affect cell proliferation both in exponentially growing cells and in overgrowth conditions. On the contrary, cells overgrowing in LRE were prone to respond to this stress by inducing autophagy instead of apoptosis, which was not observed in cells parallelly cultured in RRE.

## Materials and Methods

### Cell Lines and Proliferation Curve

The hybridoma A11 mouse cell line, which contains a pKZ1 chromosomal inversion cassette (10, 11) was used for the experiments. Cells were cultured under identical conditions in RPMI 1640 (Gibco) supplemented with 5% FBS, L-glutamine, and penicillin-streptomycin at 37°C in a 5% CO_2_ humidified incubator. For the evaluation of the role of gamma component, cells were further cultured in parallel in two different incubators, with or without an iron shield, which is able to reduce the gamma component of the radiation spectrum by a factor of about four. For each experiment, parallel cell cultures were plated at 2 × 10^4^ cells/mL in 25 mL of complete medium in 75 cm^2^ flasks and grown in quadruplicate for up to 4 weeks at the LNGS Gran Sasso underground laboratory and at the ISS, under low (LRE) and reference (RRE) environmental radiation, respectively. Cells were passaged every Monday (72 h of culture) and every Friday (96 h of culture). An aliquot of 0.5 mL of cells was diluted 1:10 with Isoton Diluent (Beckman Coulter) and counted using an automatic cell counter (Coulter Counter). At every cell passage (every 72 h and 96 h), cells were seeded at 2 × 10^4^ cells/ mL in 25 mL of complete medium in 75 cm^2^ flasks. Aliquots of the cell suspension were also used to construct a growth curve along a period of 11 days.

After 4 weeks of culture in LRE, cells were moved to the RRE laboratory and grown for 2 more weeks in RRE.

### Protein Extraction and Western Blot

Cells were centrifuged at 1,200 RPM, and protein extracts were prepared at +4°C in RIPA buffer (1x phosphate buffer, 1% NP40, 0.5% sodium deoxycholate, 0.1% SDS, 10 μL/mL PMSF, 30 μL/ml aprotinin; 10 μL/mL sodium orthovanadate) containing complete mini EDTA-free protease inhibitors (Roche Molecular Biochemicals, Mannheim, Germany). Protein concentration was determined by the BCA assay (Pierce, Thermo Fisher). Forty micrograms of proteins were loaded onto a SDS-PAGE and subjected to electrophoresis, and then proteins were electro-transferred to a nitrocellulose membrane (Whatmann, Dassel, Germany) and hybridized to anti-PARP1 (1:1,000; Cell Signaling Technology, cat #9542), anti-p53 (1:1,000; Cell Signaling Technology, cat #2524), anti-P-Ser392-p53 (1:1,000; Cell Signaling Technology, cat #9281), anti-P-Ser15-p53 (1:1,000, Cell Signaling Technology, cat #9284), anti-HSP70 (1:1,000 Cell Signaling Technology, cat #4876), anti-Caspase 3 (1:1,000, Cell Signaling Technology, cat #9662), anti-LC3B (1:1,000, Thermofisher, cat. #PA5-32254, which recognizes both LC3BI and II), anti-actin (1:2,000 Santa Cruz Biotech, cat sc-8432). Membranes were then incubated with specific horseradish peroxidase-conjugated secondary antibodies (Santa Cruz Biotech). Protein bands were visualized using a chemiluminescent detection system (Thermo Scientific, Rockford, USA).

### Densitometric Analysis and Statistics

Densitometric analysis of immunoblotting was performed by using the ImageJ software (Rasband, W.S., ImageJ, U.S. National Institutes of Health, Bethesda, Maryland, USA, https://imagej.nih.gov/ij/, 1997–2018). All densitometries were expressed as a ratio of analyzed protein to endogenous control expression. Due to the demanding and very peculiar underground working conditions, it was possible to iterate just some immunoblotting experiments (i.e., anti-P-p53 Ser392, anti-PARP-1, anti-Caspase 3) for which statistical elaboration was provided. Results were expressed as mean of two independent experiments ± SD. Data were analyzed using the GraphPad Prism (7.0 version) software and statistical analysis of the results was performed using the 2-tailed Student's *t*-test. *P* < 0.05 were considered statistically significant.

## Results

### Setting Up of Experimental Conditions

**Figure 1:**
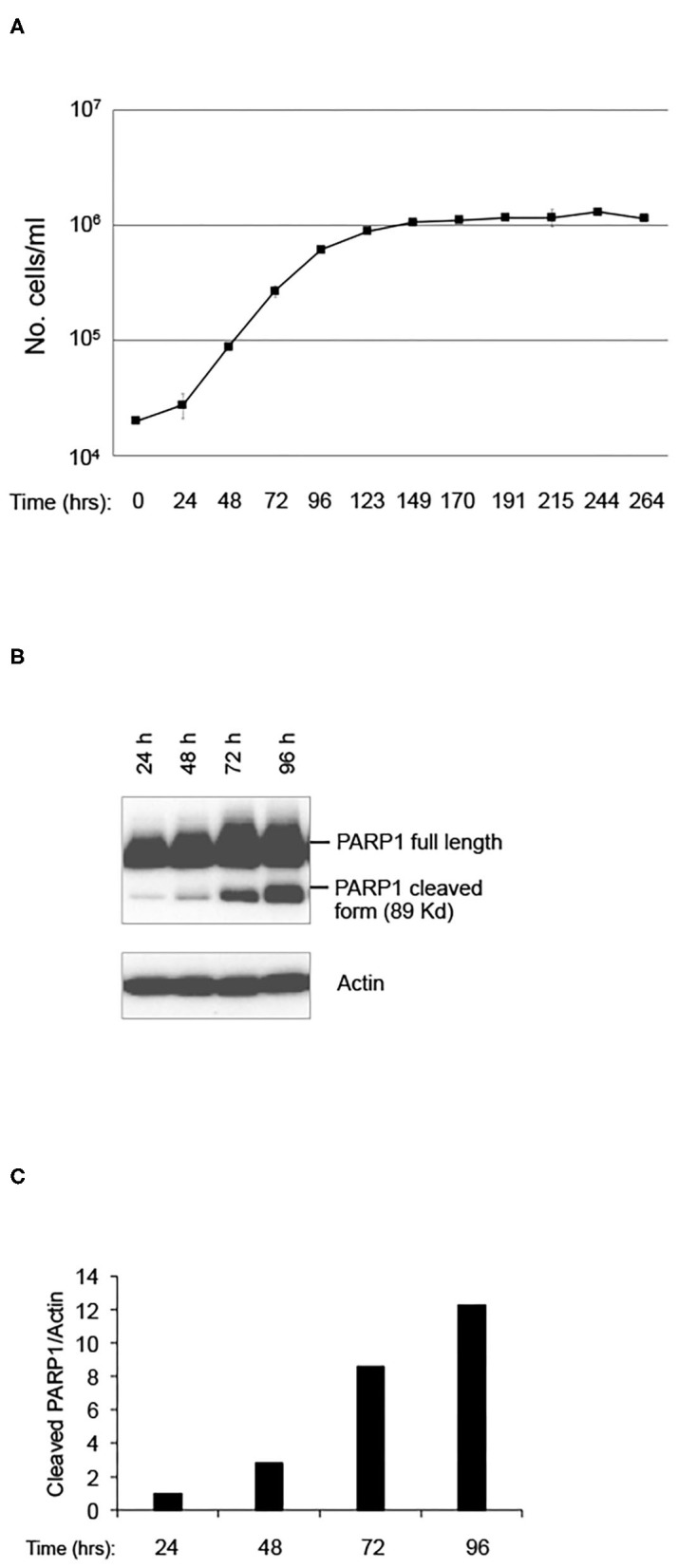
Setting up of pKZ1 A11 mouse hybridoma cells growth condition. **(A)** Proliferation curve of pKZ1 A11 mouse hybridoma cells cultured at the ISS reference laboratory in Rome. Cells were counted in triplicate. Values are means ± SD. **(B)** pKZ1 A11 mouse hybridoma cells cultured as in **(A)** and analyzed for PARP1 cleavage by western blotting. **(C)** Densitometry analysis of western blot shown in **(B)**.

In order to evaluate the effect of LRE on cell behaviors, such as cell proliferation and stress-induced cell death, a pKZ1 A11 hybridoma cell line was chosen as a model. This cell line was obtained by fusion of splenic lymphocytes of pKZ1 mouse with murine multiple myeloma cells P3653 (10). The pKZ1 mouse model has been extensively used to study the effects of low dose radiation on the adaptive response and to identify hormetic mutation responses (12–20). In order to set up the experimental conditions, pKZ1 A11 hybridoma cells were cultured in the RRE laboratory in Rome at the ISS (Figure 1A). Cell proliferation curve displayed a linear growth between 24 to 72 h, showing an inflection at 96 h and reaching a plateau at 123 h (Figure 1A). Interestingly, after 96 h, we observed a strong cleavage of PARP1, a known marker of apoptosis (Figures 1B,C) (21–23). Increase of PARP1 cleavage was detected in a time-dependent manner, reaching high level after 96 h, when cells initiated to enter to the proliferation plateau and began to stay in overgrowth stress conditions, due to nutrient and growth factor deprivation, pH changes of medium, higher oxygen need and consumption. These data indicated that, in RRE, pKZ1 A11 cells are prone to initiate and progress through apoptosis in response to the onset of overgrowth stress conditions.

### Growth of pKZ1 A11 Cells Is Not Impaired in Low Radiation Environment

**Figure 2:**
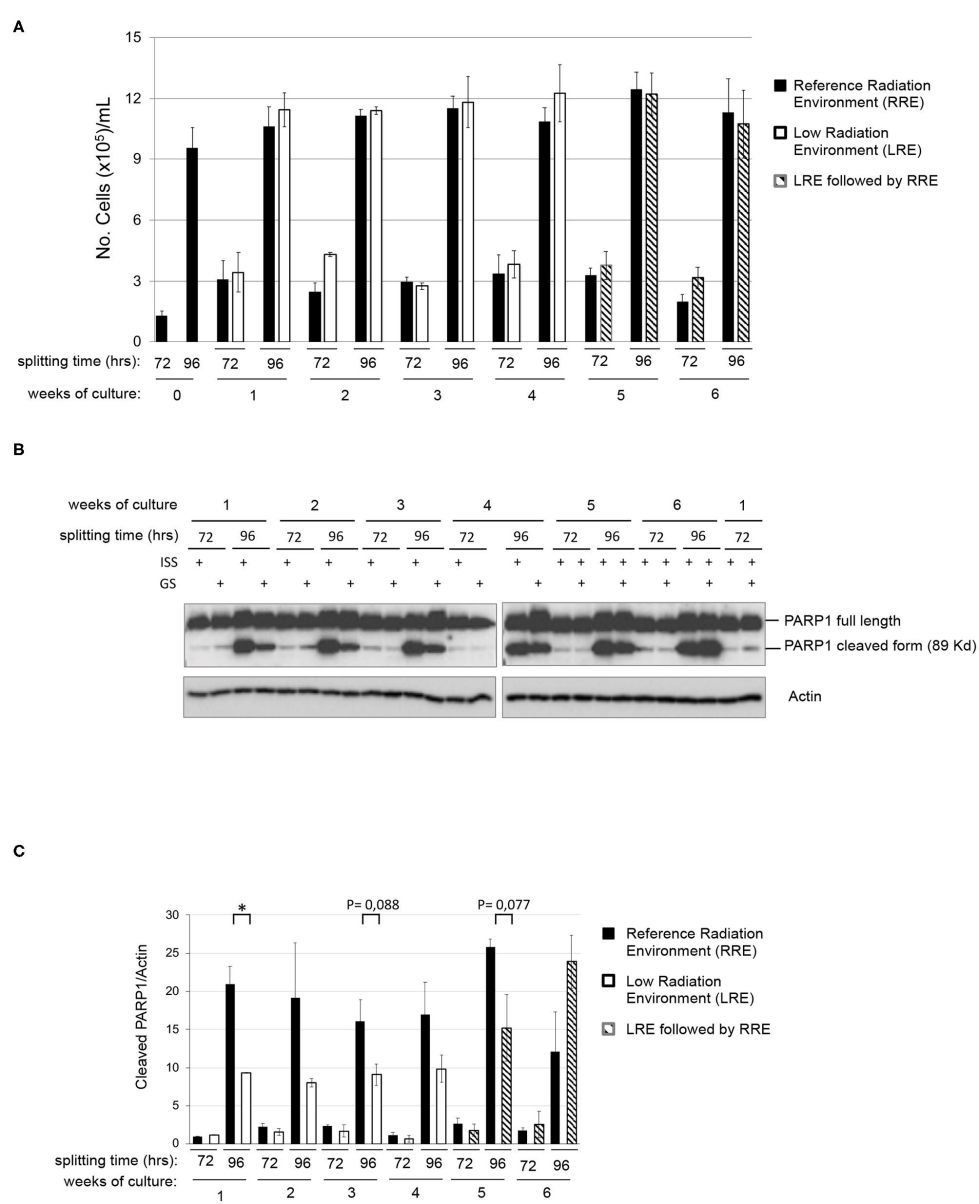
Effects of low radiation environment on pKZ1 A11 mouse hybridoma cell proliferation and overgrowth-induced apoptosis. **(A)** A twin culture of pKZ1 A11 mouse hybridoma cells was set up in quadruplicate at the ISS reference laboratory in Rome and at the Gran Sasso underground laboratory. Cells were counted and split twice a week (after 72 h and after 96 h) for 4 weeks. After 4 weeks of underground cell growth, cells were moved to the ISS-Rome reference lab and grown for additional 2 weeks (weeks 5 and 6). Each cell culture was counted in triplicate. Values are means ± SD. **(B)** Western blots showing PARP1 cleavage in pKZ1 A11 mouse hybridoma cells grown as in **(A)**. ISS, Istituto Superiore di Sanità, RRE; GS, Gran Sasso National Laboratory, LRE. **(C)** Densitometric analysis of anti-PARP1 western blots. Values are means of two independent experiments ± SD. *P < 0.05.

In order to assess the existence of differential behaviors potentially due to the environmental radiation levels, a twin culture of pKZ1 A11 cells was maintained in parallel in LRE and RRE, exactly under the same conditions, for 4 weeks and split twice a week, every 72 and 96 h. As shown in Figure 2A, no differences were observed in terms of proliferation rate in such conditions between cells grown in RRE and LRE. In accordance with data shown in Figures 1A,B, cell count at 72 h of each week of culture displayed a number of cells consistent with a linear growth rate, and cell count at 96 h of each week of culture showed a number of cells consistent with an initial overgrowth plateau state (Figure 2A). Moreover, to evaluate the effect of RRE on cells cultured for 1 month in LRE, pKZ1 A11 cells grown for 4 weeks in LRE were moved to the RRE laboratory and cultured for two more weeks (Figure 2A, weeks 5 and 6). No differences in proliferation were observed. These data showed that LRE does not influence the cell growth behavior of pKZ1 A11 cells.

### PARP1 Cleavage Is Strongly Reduced in LRE-Overgrown Cells

To evaluate the effect of LRE at the molecular levels, cells grown as described in Figure 2A were collected twice a week (after 72 and 96 h of culture) for 6 weeks and analyzed for PARP1 cleavage. As expected, levels of PARP1 cleavage increased in a time-dependent manner, consistent with that described above (Figure 1B). At the same way, similar behavior was detected in terms of PARP1 expression and cleavage in RRE and LRE under conditions of linear growth (72 h). Interestingly, lower levels of PARP1 cleaved form were detected in LRE overgrown cells (96 h) compared to those cultured in RRE (Figures 2B,C). PARP1 cleaved protein was reduced approximately by half in LRE compared to RRE (Figure 2C). Interestingly, this effect globally reverted when, after 4 weeks of underground culture, cells were moved to the RRE laboratories for 2 more weeks (Figures 2B,C, weeks 5 and 6), indicating a plasticity of cells toward their response to low environmental radiation.

### Low Radiation Environment Switches pKZ1 A11 Mouse Hybridoma Response to Overgrowth From Apoptosis Toward Autophagy

**Figure 3:**
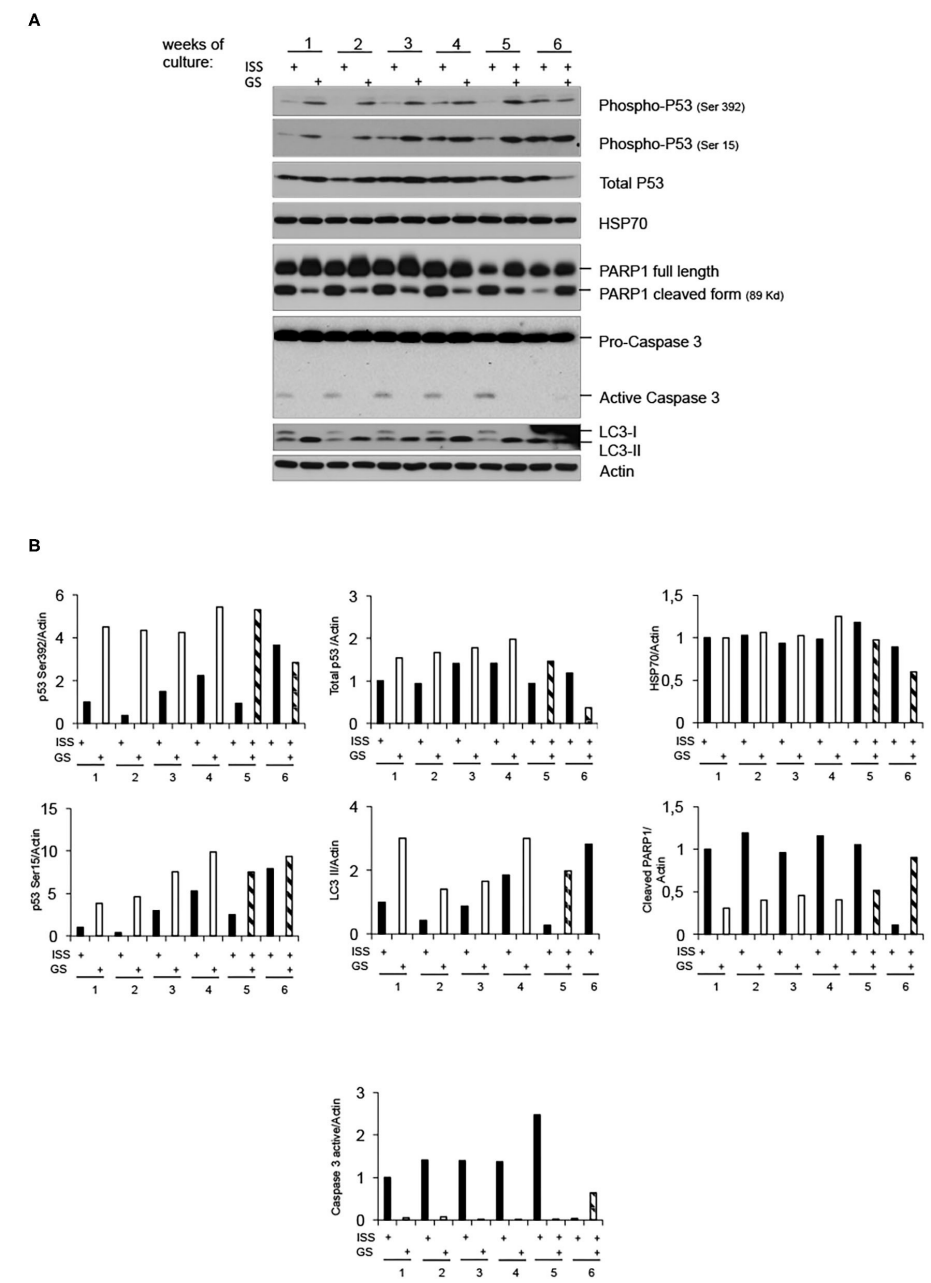
Low radiation environment switches pKZ1 A11 mouse hybridoma overgrowth cell response from apoptosis toward autophagy. **(A)** Western blots showing activation of p53 and induction of LC3B-II in LRE (LNGS)-grown pKZ1 A11 mouse hybridoma cells and activation of PARP1 and caspase 3 in RRE (ISS) grown pKZ1 A11 mouse hybridoma cells. **(B)** Densitometry analysis of western blots shown in **(A)**.

To further shed light on the molecular response of overgrown cells cultured in LRE vs. RRE, we performed the western blot analysis of proteins known to be implicated in cell stress response, such as Heat Shock Protein (HSP) 70; genomic stability control and cell death regulation, such as p53; markers of apoptosis, such as caspase 3; and markers of autophagy, such as LC3B. As shown in Figure 3, HSP70 levels did not differ between LRE- and RRE-cultured cells upon stress stimulation due to overgrowth (96 h of culture). As expected, the total p53 levels were not significantly modulated, whereas the active form of p53, evaluated as phosphorylation in both Ser15 and Ser392, was strongly induced in LRE overgrowth conditions. A canonical function of p53 is the induction of apoptotic response when cells cannot efficiently repair acquired DNA damage (24, 25). In addition to this function, p53 has been shown to be a modulator of the autophagic process (26), which is known to promote survival upon starvation stress and maintain metabolic homeostasis through degradation and recycling of intracellular components. Literature showed that autophagy suppresses p53 and, also, p53 activates autophagy as part of its protective function (26). In our experimental settings, expression of active p53 correlated with the conversion of LC3B-I to the lower migrating form LC3B-II which is used as an indicator of autophagy especially in condition of nutrient starvation (27, 28), showing that, in LRE conditions, the cell response to overgrowth stress is to switch toward autophagy (Figure 3). Finally, in accordance with PARP1 cleavage data (Figures 2B, 3), apoptotic cell induction in RRE overgrowing cells was confirmed by caspase 3 induction. These data suggest an alternative mechanism of response and defense toward stress/damage induced by long-term cell culture in strongly reduced radiation background.

### Gamma Component of Radiation Does Not Affect LRE Overgrowth Cell Response

**Figure 4:**
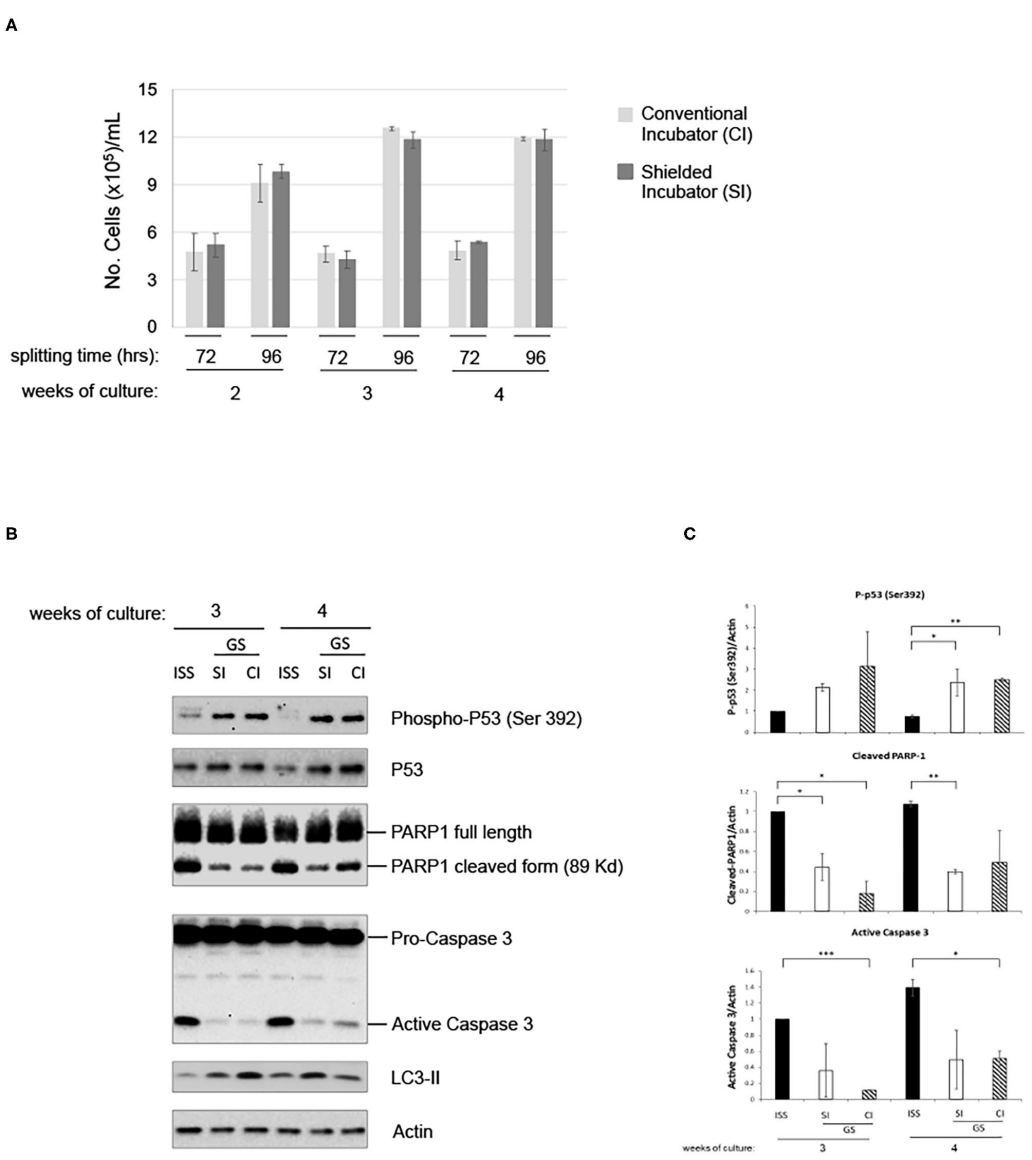
Gamma radiation does not influence pKZ1 A11 mouse hybridoma cell response to overgrowth in low radiation environment. **(A)** Three cultures of pKZ1 A11 mouse hybridoma cells were set-up in quadruplicate and grown at the ISS reference laboratory in Rome (ISS) and at the Gran Sasso underground laboratory (GS) in a conventional incubator (CI) and in a γ-radiation shielded incubator (SI). Cells were counted and split twice a week (after 72 h and after 96 h) for 4 weeks. Cells were counted in triplicate. Values are means ± SD. **(B)** Cells grown as in **(A)** were collected after 3 and 4 weeks of cultures (at 96 h splitting time) and analyzed for apoptotic and autophagic markers by western blots. **(C)** Densitometric analysis of anti-P-p53 (Ser392), anti-PARP1, anti-caspase3 western blots. Values are means of two different experiments ± SD. *P < 0.05, **P < 0.005, ***P < 0.0005.

To analyze the putative contribution of gamma component in inducing the above-described effects, cells were further grown in parallel in the LRE laboratory in two incubators, with or without iron shield. The iron (Fe) shield can reduce the gamma component of the radiation spectrum by a factor of about four. As shown in Figure 4A, proliferation of pKZ1 A11 cells was very similar in both conditions. In addition, in presence or absence of iron shielding, a globally comparable protein expression of phospho-p53, PARP1 cleavage and caspase 3 activation, and LC3-II induction was observed (Figures 4B,C), suggesting a negligible role of the gamma component in such cell responses and behavior.

## Discussion

In the last two decades, several efforts have been made by researchers to clarify the roles of low environmental radiations on cell response to stress stimuli. The linear no-threshold (LNT) model was first used in the radioprotection field to predict cell and DNA damage induced by ionizing radiation (Ann. ICRP, 2007). Several studies have, indeed, shown that risk assessment for low radiation dose does not follow a linear curve (29). Interestingly, Non-Target Effects (NTEs) are particularly evident at low doses and include the role of cell communication and responses at the tissue and systemic levels. Among the NTEs, it is possible to highlight the genomic instability and the Bystander Effect (BE), which seem to have a role in increasing the risk of developing cancer above the estimates made by extrapolation of the LNT model, and the Adaptive Response (AR), which, on the contrary, would be a protection against the development of cancer (30). In any case, a common feature of these phenomena is, however, the lack of dose linearity (31). Therefore, shedding light on the molecular mechanisms underlying cell response to low doses of radiation is particularly relevant in the radioprotection field.

In this work, we showed that cells grown in a LRE at the underground Gran Sasso National Laboratory (LNGS) of the Italian Institute of Nuclear Physics (INFN) display a qualitatively different response to stress induced by overgrowth, condition characterized by nutrient and growth factor deprivation, pH changes of medium, higher oxygen need and consumption. We did not find any difference in the growth of cells cultured in LRE compared to cells cultured in the RRE laboratory. On the other hand, after 96 h of growth in RRE, we noted an increase of PARP1 cleavage. This was described as an early marker of apoptosis (21). Interestingly, we observed a switch from apoptosis toward autophagy in LRE cultured cells, which appears to be mediated by p53.

Autophagy is an evolutionarily conserved catabolic pathway involved in many physiological and pathological mechanisms. It induces lysosomal degradation and recycling of non-functional cytoplasm components or organelles, generating substrates which promote stress adaptation and survival. However, autophagy is not intended as just a protective mechanism, as it can lead to an autophagy-associated and autophagy-mediated death, where autophagy occurs by accompanying or inducing apoptosis, or to an autophagy-dependent cell death, which is independent of both apoptosis and necrosis processes (32–34). Nevertheless, molecular mechanisms at the base of connections between autophagy, cell death, and cell fate decision are still unrevealed. Autophagy is increased by cell stress (e.g., DNA damage, hypoxia, starvation, or growth factor deprivation) and depends on complicated signaling networks which include mediators of relevant cell responses, such as p53, NF-κB, and STAT3 (35). It has been described that, in cancer, autophagy provides a sort of self-eating process to recruit nutrients in a condition where they become unavailable (36). Several studies indicate that stress can induce autophagy through two ways: an early response which takes place within minutes to hours and is essentially mediated by protein post-translational modifications, and a second, delayed response, which is based on specific activation of transcription (37). Moreover, an interesting relationship between p53 and autophagy has been reported, based on the use of *in vitro* and *in vivo* preclinical models (26): whereby autophagy can suppress p53 and p53 can activate autophagy. The tumor suppressor p53 usually acts as a tetrameric nuclear transcription factor, inducing genes coding for proteins involved in cell cycle arrest, apoptosis, or autophagy. On the other hand, it was described that cytoplasmic p53 can also promote cell death at the mitochondrial level and suppress autophagy (38). Furthermore, under stress conditions, p53 undergoes post-translation modifications able to avoid mdm-2-driven proteasomal degradation or, alternatively, mdm-2 can be degraded or sequestered by ARF, thus inhibiting the autophagic process. Ser392 is one of the highly conserved phospho-acceptor sites involved in p53 tumor-suppressor activities by enhancing p53 tetramers and DNA binding stabilization (39). Ser392 phosphorylation occurs not only after UV damage, but also after a range of different stimuli, such as etoposide, mdm-2 inhibitors or ionizing radiations (40). Since p53 is a well-known regulator of the apoptotic process too, further experiments will be needed to clarify, in particular, the specific signaling pathway and mechanisms through which p53 induces autophagy instead of apoptosis in our experimental settings.

Data here reported highlight different responses of cells reaching overgrowth stress conditions in RRE or LRE, respectively leading to apoptosis or autophagy, and characterized by the induction of specific mediators/markers involved in those pathways. Those novel results open to further investigations to examine in depth the molecular functional mechanisms specifically activated in RRE or LRE.

In particular, our findings draw attention to a p53-mediated autophagic cell response in a condition of almost absence of environmental radiation (Gran Sasso National Laboratory) instead of the apoptotic response observed in normal radiation environment. This is particularly fascinating and leaves to hypothesize that environmental radiation levels at the Earth's surface might represent a constitutive low-level stress able to sensitize cells to a prompt response in case of different acute stresses, which could produce further propagation of DNA damage in the case of inefficiently induced apoptosis.

Of further interest is to understand the role of both low and high LET components, both present in the external environment, in determining the response of the biological system. To answer this question, experiments are ongoing at the LNGS, presently focused on the role of the gamma component in the biological response(s) of fruit flies.

## Data Availability Statement

The raw data supporting the conclusions of this article will be made available by the authors, without undue reservation.

## Author Contributions

MF, DVer, DVec, and DC performed the LRE experiments. EF and GE performed the RRE experiments. BD performed the western blot. MB and LI contributed to LRE experiments' setup. PS and LS conceived the study. AT analyzed the data, wrote, and revised the manuscript. FZ, MT, and EA conceived the study, elaborated data, and wrote and revised the manuscript. All authors contributed to the article and approved the submitted version.

## Conflict of Interest

The authors declare that the research was conducted in the absence of any commercial or financial relationships that could be construed as a potential conflict of interest.
